# Mechanical Properties of 3D-Printed PEEK/HA Composite Filaments

**DOI:** 10.3390/polym14204293

**Published:** 2022-10-12

**Authors:** Jianfeng Kang, Jibao Zheng, Yijun Hui, Dichen Li

**Affiliations:** 1 Jihua Laboratory, Additive Manufacturing Institute, Foshan 528200, China; 2 State Key Laboratory for Manufacturing Systems Engineering, School of Mechanical Engineering, Xi’an Jiaotong University, Xi’an 710032, China; 3 National Medical Products Administration (NMPA) Key Laboratory for Research and Evaluation of Additive Manufacturing Medical Devices, Xi’an Jiaotong University, Xi’an 710032, China

**Keywords:** PEEK/HA composite, 3D-printed filament, mechanical property, anisotropy, finite element analysis

## Abstract

The incorporation of bioactive ceramic into polyether ether ketone (PEEK) was expected to improve the bioinertia and hydrophobicity of pure PEEK, further facilitating osseointegration and bone ingrowth. However, the addition of bioceramic also changes the anisotropy of mechanical properties and failure mechanism of composite. Therefore, three-dimensional printed (3D-printed) PEEK/hydroxyapatite (HA) composite filaments with differing proportions (HA content: 10–30 wt%) were prepared using physical mixture and melting extrusion processes. The tensile elastic modulus and tensile strength of composite filaments were tested experimentally. These microscopic models, with multiple diameter variations and differing dispersity of HA particles, were built to estimate mechanical properties using finite element analysis. Based on a generalized version of Hooke’s Law, the influence of diameter variation and particle clustering on the elastic modulus was evaluated. The mathematical relationship between the elastic modulus and volume fraction of the bioceramic was established using the Halpin–Tsai model. The results showed that with an increase in HA content from 10 wt% to 30 wt%, the elastic modulus of the composite increased from 2.36 GPa to 2.79 GPa, tensile strength decreased from 95 MPa to 74 MPa, and fracture elongation decreased from 63% to 23%, presenting brittle fracture failure. When the dispersion of particles was uniform, the elastic modulus was less affected by diameter variation, but the modulus anisotropic coefficient was greatly affected by the composition ratio, particle diameter, and dispersity. Hence, 3D-printed PEEK/HA composite filaments can meet the strength requirements of human bone, and understanding the influence of mechanical anisotropy plays a very important role in the design, manufacture, and clinical application of medical implants.

## 1. Introduction

Polyether ether ketone (PEEK), a material with excellent biocompatibility and anti-corrosion, similar density and mechanical properties compared to natural bone tissue, and high radiographic penetrability, has been widely used to fabricate medical protheses using additive manufacturing technology, such as thoracic or rib prosthesis [1,2], intervertebral fusion cages [3], skull prosthesis [4], mandibular prothesis [5], etc. However, the bioinertia and hydrophobicity of pure PEEK material make creating a strong interface with surrounding soft or hard tissue and the induction of tissue ingrowth difficult [6]. Hence, improving the bioactivity of PEEK material plays a key role in the functionalized repair and stability of medical prostheses.

Currently, modification strategies for PEEK material mainly include surface modification and material hybrid modification. For surface modification, physical or chemical treatments have often been used to improve the surface roughness or microporous structure of material, as well as the deposition of bioactive materials to alter bioactivity and osseointegration [7]. The surface modification layer generally belonged to the microscopic scale and exhibited a risk of peeling off from the PEEK matrix under the condition of a complex loading environment, owing to the weak interfacial bonding strength [6]. These detached particles reduce the bioactivity of the substrate, while leading to an inflammatory response [8]. Therefore, some studies have turned to the development of bioactive PEEK-based composite materials by the addition of bioactive ceramics into the PEEK matrix, such as hydroxyapatite (HA) [9,10], β-tricalcium phosphate [11], calcium silicate [12], bioglass [6], etc. The superior bioactivity of PEEK/bioceramic composites in comparison to pure PEEK material has been confirmed by cellular and animal experiments [13,14], which may provide an effective way of obtaining both mechanical and biological benefits.

Despite an improvement in bioactivity, the addition of bioceramics also affects the overall mechanical properties of the composites to some extent. Zheng et al. [15] fabricated 3D-printed filaments of PEEK/HA composite with different mass fractions by using physical mixing and melt extrusion processes, and evaluated the mechanical properties of the resulting 3D-printed samples. Compared with the mechanical properties of pure PEEK, the tensile modulus of PEEK/HA composite increased by 68.6% with the increase in HA content to 30 wt%, while tensile strength decreased by 48.2%. Manzoor et al. [13] investigated the effect of material formulation on the mechanical performance of 3D-printed PEEK/HA composites, and a small reduction in ultimate tensile strength (~14%) and Young’s modulus (~5%) in PEEK-based filaments containing 10 wt% of pure nano-HA was observed in comparison to pure PEEK. Rodzen et al. [16] evaluated the tensile and flexural properties of 3D-printed PEEK/HA composites with differing HA contents (0–30 wt%), and pointed out that the tensile and flexural modulus significantly increased with the additional HA material, while the variation in tensile and flexural strength was irregular. After comparing the above studies, it can be observed that the difference in mechanical properties of the 3D-printed PEEK/HA samples was relatively significant, even with the same amount of additional HA material. To some extent, it may be affected by the preparation of composite filaments and the 3D-printing process. However, the influence of size and agglomeration of additive particles on the mechanical properties and anisotropy have rarely been analyzed.

Hence, this study fabricated 3D-printed filaments of PEEK/HA composite with differing HA contents. According to the distribution of particle diameter observed using a scanning electron microscope, a numerical model was established to analyze the mechanical properties of the composite. The effects of material proportion, particle diameter variation and dispersion uniformity on mechanical properties were investigated. Tensile testing of PEEK/HA composite filaments was performed. Finally, the mathematical relationship between elastic modulus or tensile strength and volume fraction of HA was built. Through comprehensive analysis of mechanical properties and anisotropy of PEEK/HA composite filaments, this study lays a solid foundation for the design and evaluation of 3D-printed PEEK/HA composite medical implants.

## 2. Materials and Methods

### 2.1. Fabrication of PEEK/HA Composite Filament

**Figure 1:**
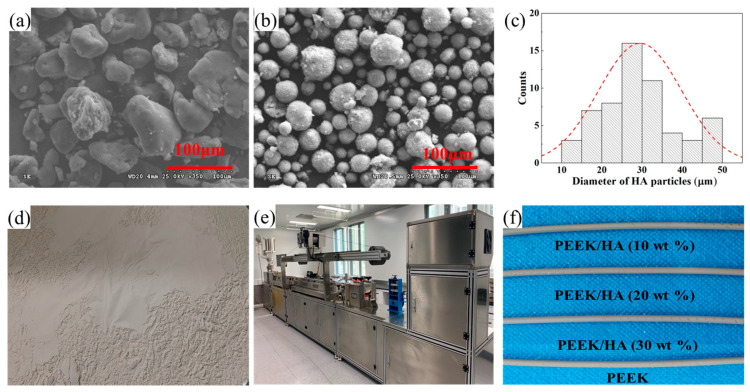
Fabrication of PEEK/HA composite filaments: microtopography of PEEK (**a**) and HA (**b**); (**c**) size distribution of HA particles; (**d**) PEEK/HA (20 wt%) mixture; (**e**) twin-screw extruder; (**f**) filament samples.

PEEK powder (150 PF, Victrex, Thornton Cleveleys, UK) and HA powder (HAP-08, Emperor, Nanjing, China) were used to fabricate the PEEK/HA composite. The microscopic morphology of two powders was observed using a scanning electron microscope (SU-8010, Hitachi, Tokyo, Japan). As shown in Figure 1a–c, PEEK powder exhibited an irregular shape, while HA could be regarded as a spherical particle with a diameter of 10–50 μm. These powders were dried in a furnace at 150 °C for 4 h before being premixed in various compositions. PEEK/HA mixtures (in Figure 1d) with HA contents of 10, 20, and 30 wt% were prepared by a V-type mixer at a rotation speed of 60 rpm for 8 h. Among the dry mixing processes, fifty spherical quartz grinding balls with a diameter of 15 mm were added to the PEEK/HA mixtures for grinding and stirring. As shown in Figure 1e, a co-rotating twin-screw extruder (FLD35, ACC Machine, Suzhou, China) was utilized to compound the PEEK/HA composite filaments with a diameter of 1.75 mm. It was operated at a screw speed of 45 rpm and an exact melt temperature of 370 °C. The composite filaments with differing HA contents are shown in Figure 1f.

### 2.2. Mechanical Testing of Composite Filaments

According to the ASTM D4018–11 standard, the tensile testing of PEEK filament and PEEK/HA composite filament was performed using a universal testing machine (CMT4304, MTS Corp., Eden Prairie, MN, USA) at room temperature. Both ends of each filament specimen with a length of 250 mm were adhered to cardboard for a length of 50 mm. A crosshead separation rate of 2 mm/min was set, and an extensometer with a gauge length of 100 mm was used to measure deformation. Thereafter, tensile elastic modulus and tensile strength were calculated. For each group, four duplicate specimens were tested to ensure repeatability. Finally, the fracture surface of composite filaments was observed by SEM.

### 2.3. Analysis of Mechanical Properties

According to the generalized Hooke’s Law, the tensor form of stress–strain relationship during the elastic deformation stage can be expressed as follows:

(1) $$\sigma_{ij}=C_{ijkl}\varepsilon_{kl}$$

where ***C****_ijkl_* is a fourth-order stiffness matrix with 81 components. Owing to the inherent symmetries, the coefficients of the stiffness matrix can be reduced to 21, and the matrix format is given as Equation (2).


(2) $$\left[\begin{array}{c} \sigma_{11}\\ \sigma_{22}\\ \sigma_{33}\\ \sigma_{12}\\ \sigma_{13}\\ \sigma_{23} \end{array}\right]=\left[\begin{array}{cccccc} C_{11} & C_{12} & C_{13} & C_{14} & C_{15} & C_{16}\\ & C_{22} & C_{23} & C_{24} & C_{25} & C_{26}\\ & & C_{33} & C_{34} & C_{35} & C_{36}\\ & & & C_{44} & C_{45} & C_{45}\\ & & & & C_{55} & C_{56}\\ & & & & & C_{66} \end{array}\right]\left[\begin{array}{c} \sigma_{11}\\ \sigma_{22}\\ \sigma_{33}\\ \sigma_{12}\\ \sigma_{13}\\ \sigma_{23} \end{array}\right]$$

In order to calculate each stiffness coefficient, different loading and boundary conditions were set using a multi-level hypothesis. The specific solution process was mentioned in our previous study [17]. The spatial distribution of elastic modulus for PEEK/HA composite can be plotted by the transformation of polar coordinates and programing using Matlab software (Version R2012 b, MathWorks Inc., Natick, MA, USA). Meanwhile, the anisotropy of elastic modulus was also further investigated quantitatively using the following equation:

(3) $$f_{DA}=2C_{44}/\left(C_{11}-C_{12}\right)$$

**Figure 2:**
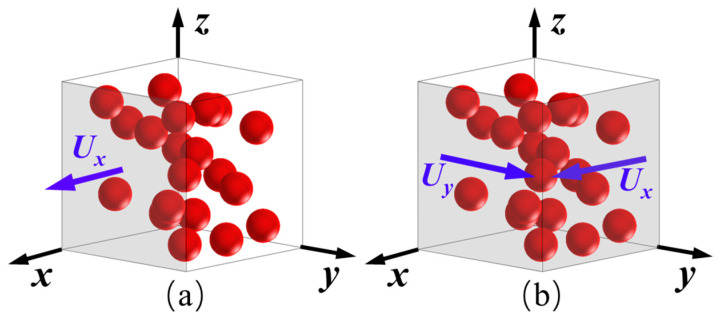
Boundary conditions of calculation model for mechanical anisotropy of composite: (**a**) normal strain; (**b**) shear strain.

A micro-geometrical model of the PEEK/HA composite was built by randomly distributing spherical HA particles into the PEEK matrix. Next, the geometrical model was imported into Abaqus software (Version 6.14, Dassault systems, Vélizy-Villacoublay, France). The material properties of each component of PEEK/HA composite were set, respectively, with an elastic modulus of 1.97 GPa and a Poisson ratio of 0.3 for the PEEK matrix, and corresponding parameters of 100 GPa and 0.28 for HA particles [18]. According to the diameter of HA particles, three element sizes of 0.0025 mm, 0.005 mm, and 0.01 mm were used to analyze the meshing sensitivity to eliminate the effect of meshing size, and a relative deviation of less than 5% for the elastic modulus was achieved. Finally, an element size of 0.005 mm was chosen. The contact interface between PEEK and HA was set as shared nodes and “Tie” property. As shown in Figure 2, the normal strain and shear strain in different directions were applied, respectively, and the corresponding stiffness coefficient was calculated according to the displacement and output reaction force.

**Figure 3:**
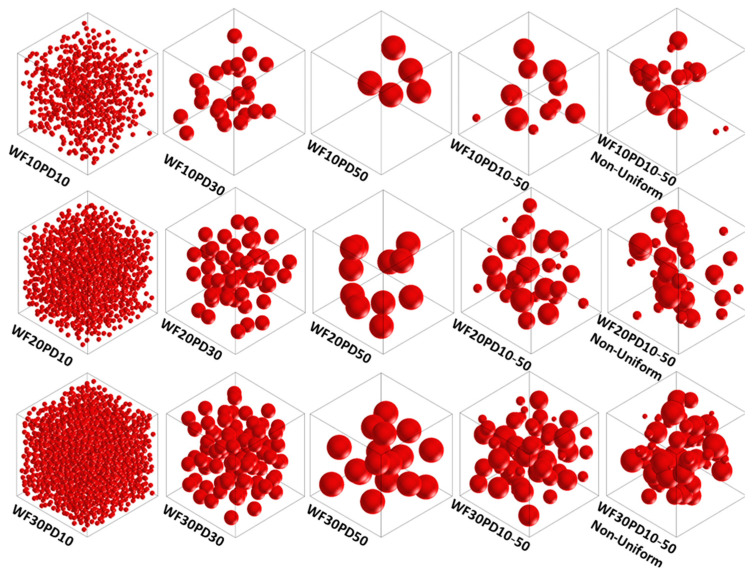
Micromodel of composites with differing composition proportions (WTx), particle diameters (PDx) and dispersibilities (uniform distribution by default, non-uniform distribution is marked).

Based on the above analysis method, three kinds of PEEK/HA composites with HA contents of 10, 20, and 30 wt% were analyzed according to the fabrication of the composite filament. As shown in Figure 3, 15 models were built to investigate the influence of uniform-diameter of particles (10 μm, 30 μm, 50 μm), non-uniform-diameter particles (from 10 μm to 50 μm), and inhomogeneous particles with agglomeration on the elastic modulus. Considering the distribution randomness of HA particles, each type of model calculation was repeated 4 times by regenerating a geometric model. Through finite element analysis, the calculated results of each microscopic model were extracted to estimate elastic modulus and anisotropy, and the stress distribution laws of different components in the composite were evaluated.

## 3. Results and Discussion

### 3.1. Tensile Properties of the Composite Filaments

**Figure 4:**
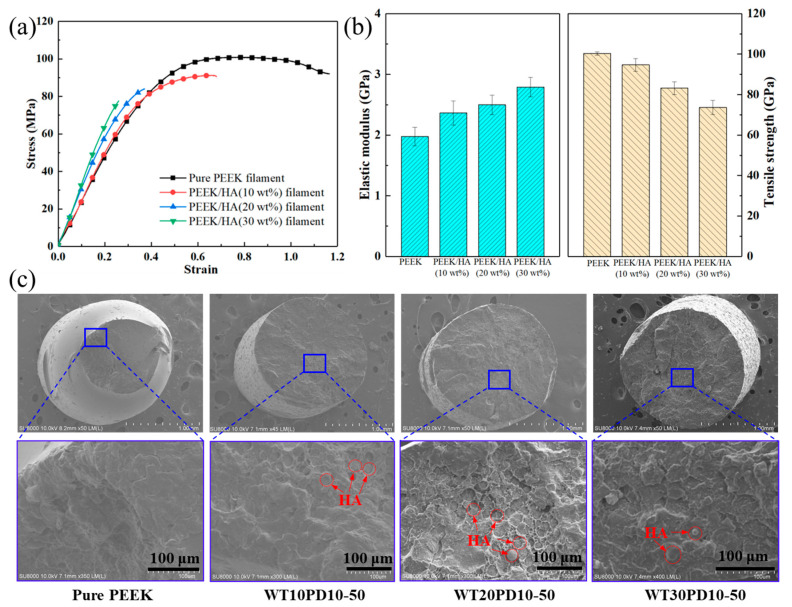
Tensile mechanical properties of composite filaments: (**a**) stress–strain curve; (**b**) elastic modulus and tensile strength; (**c**) SEM of fracture surface.

The tensile mechanical properties of PEEK and PEEK/HA composite filaments are given in Figure 4. From the stress-strain curve, the elastic modulus of composite filaments increased with the incorporation of HA, yet the tensile strength and elongation at break decreased. Thereinto, the elastic modulus and tensile strength of pure PEEK filament were 1.97 ± 0.15 GPa and 100 ± 0.8 MPa, respectively. Compared to the tensile properties of the dumbbell-shaped specimens fabricated by 3D printing or injection molding, the elastic modulus of pure PEEK filament was close to the 2.1–2.2 GPa value from previous studies [15,19], but lower than the typical value (3–4 GPa) [19]. These differences may have resulted from the effect of temperature change on the crystallinity of PEEK materials and the different process parameter settings during sample preparation. Related studies [20,21] have indicated that PEEK and its low-crystallinity composite materials have low elastic modulus and strength, exhibiting excellent toughness.

For the PEEK/HA composite filaments, when HA content increased from 10 wt% to 30 wt%, the elastic modulus increased from 2.36 ± 0.20 GPa to 2.79 ± 0.16 GPa, while the ultimate tensile strength decreased from 95 ± 3.18 MPa to 74 ± 3.46 MPa, and the percentage of total extension at fracture decreased from 63% to 23%. According to Rodzen’s study [16], under the condition of equivalent HA content, the ranges of tensile elastic modulus and ultimate tensile strength for the PEEK/HA composite specimens were 4.716–6.110 GPa and 94.2–84.9 MPa, respectively, and the percentage of total extension at fracture was between 1.6 and 2.8%. Despite similar tensile strength after comparison, the elastic modulus and the percentage of total extension at fracture were quite different. This may be due to the preparation of the composite and the effect of crystallinity. In the composite preparation process, mechanical properties were significantly influenced by the uniformity of different particle sizes and dispersions [22,23], which was the starting point of this study. Meanwhile, by performing differential scanning calorimetry analysis, the crystallinity of PEEK/HA composite from Rodzen’s study was more than 30%, which increased the elastic modulus and tensile strength, decreasing the percentage of total extension at fracture. In our previous study, the crystallinity of composite filaments varied from 15.58% to 20.7% [15]. Although the presence of HA does not adversely affect the degree of crystallinity of the PEEK component in the mixtures [13,21], the differing crystallinity of the PEEK matrix will also affect the overall mechanical properties of the composite.

After observing the fracture surface by SEM, the failure mechanism of PEEK/HA composites changed from a ductile fracture to a brittle fracture. This is mainly attributed to the change in continuous stress distribution inside the composite, owing to the random dispersion of discrete ceramic particles in the PEEK matrix. In particular, some of the local stress concentrations at the contact interface of different phases further aggravated the initiation and propagation of microcracks, which finally evolved into brittle fractures. Comparing the strength of cortical bone in different locations and orientations, usually ranging from 50–151 MPa [24], the mechanical properties of PEEK/HA composite filaments were within this range and can be selected according to the needs of practical clinical application, so as to ensure that the implant has good safety and bone growth characteristics.

### 3.2. Influence Analysis of Mechanical Properties

**Figure 5:**
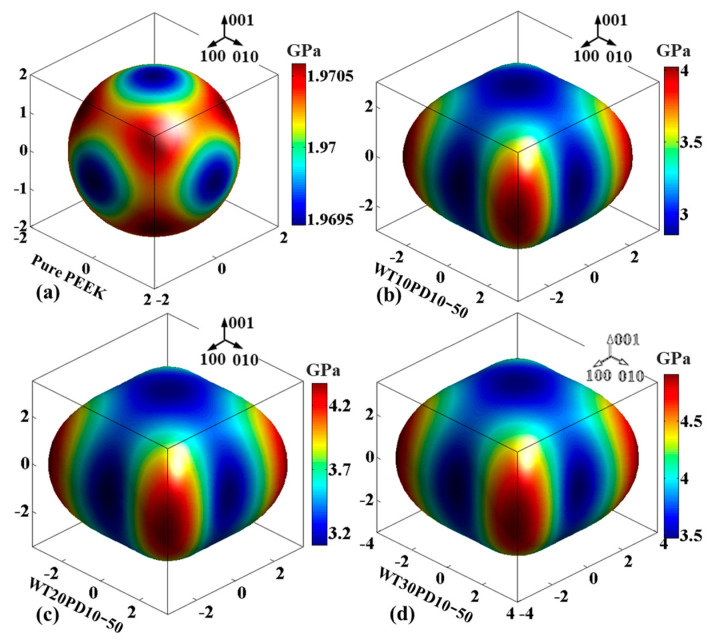
Spatial distribution of elastic modulus for the PEEK material and PEEK/HA composite: (**a**) pure PEEK; (**b**) PEEK/HA (10 wt%) composite; (**c**) PEEK/HA (20 wt%) composite; (**d**) PEEK/HA (30 wt%) composite.

The spatial distributions of elastic moduli derived from numerical analysis of the microscopic model for the PEEK matrix and PEEK/HA composites are shown in Figure 5. The distribution of the elastic modulus for pure PEEK material was approximately spherical, namely isotropic. Compared to pure PEEK material, the distribution shape of the composite’s modulus changed, owing to the incorporation of HA particles. However, with differing proportions of HA addition, the overall pattern of modulus distribution for PEEK/HA composite remained similar. Thereinto, the minimum and maximum of the elastic moduli were located in the axial and diagonal directions, respectively. These two values increased with the increase in HA content, and the ratio of *E*_min_/*E*_max_ fell between 0.6 and 0.7 within the set proportion range.

**Figure 6:**
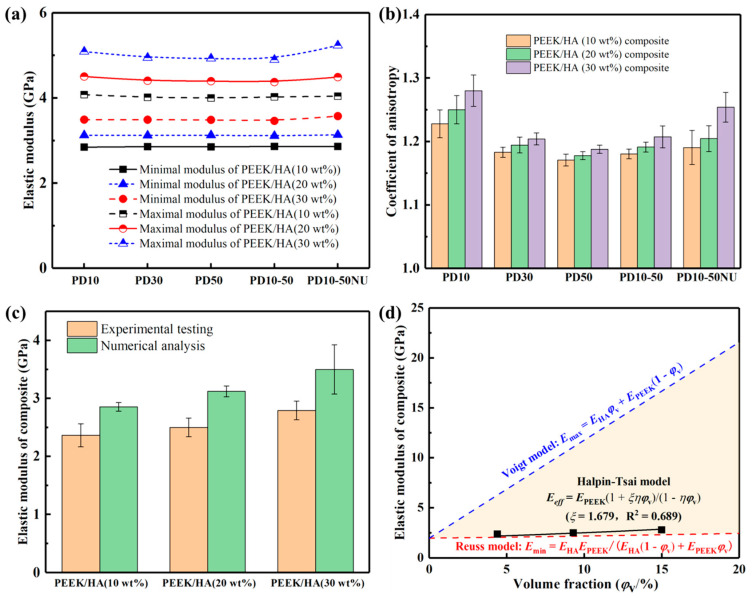
The elastic moduli of PEEK/HA composites: (**a**,**b**) The influence of particle diameter and dispersion on the modulus and anisotropic coefficient; (**c**) The comparison of elastic moduli between experimental testing and numerical analysis; (**d**) The mathematical model for effective elastic moduli of PEEK/HA composites; PD 10–50 NU denotes nonuniform distribution of HA particles with a diameter of 10–50 μm.

The influence of particle diameter variation and dispersion uniformity on the elastic modulus and anisotropy for PEEK/HA composites with differing proportions were plotted in Figure 6a,b. It can be seen that when the HA particles were distributed uniformly, the variation range between the minimum and maximum moduli was less than 5% with the increase in particle diameter, and the modulus anisotropy decreased with the increase in particle diameter. These results indicate that the influence of particle diameter on the elastic modulus of the PEEK/HA composite was relatively small under the condition of uniform particle distribution, and the increase in particle size effectively alleviates modulus anisotropy. However, in the actual composite preparation process, there are often non-uniform particle diameters, and it is difficult to avoid agglomeration. As particle agglomeration occurs, it increases the elastic modulus and its anisotropy, especially with the high HA content. Therefore, during the preparation process of PEEK/HA composite filaments, it can be considered to improve the consistency of particle size by repeated grinding, filtration and screening, and fully stirring to reduce agglomeration as much as possible, which is beneficial to ensure the stability of mechanical properties.

Moreover, the numerical analysis results were generally larger than the experimental results, with a deviation of about 20%. This difference may have resulted from the limitation of finite element analysis, causing difficulty in fully reflecting some problems in the actual preparation of composite filaments, such as the irregularity of micropore formation, particle dispersion uniformity or agglomeration, imperfect or interfacial bonding, etc. In order to establish a mathematical relationship between the elastic modulus of the composite and its internal composition, according to the Voigt model by assuming equal strain for longitudinal loading and the Reuss model with equal stress for transverse loading [25], the upper and lower bound for the elastic modulus of composite can be calculated by Equations (4) and (5). In fact, due to the effects of particle dispersion uniformity, interfacial binding, and internal defects in the preparation process, the ideal situation of equal stress or strain was difficult to achieve, which resulted in the predicted value of the Voigt or Reuss models differing greatly from the experimental result [26]. Herein, the Halpin-Tsai model was used to develop generalized equations with empirical terms, providing a better estimate of the effective properties, as shown in Equation (6).

(4) $$E_{Voigt}=\varphi_{V}E_{\mathrm{HA}}+\left(1-\varphi_{V}\right)E_{\mathrm{PEEK}}$$

(5) $$E_{Reuss}=E_{\mathrm{HA}}E_{\mathrm{PEEK}}/\left(E_{\mathrm{HA}}\left(1-\varphi_{V}\right)+E_{\mathrm{PEEK}}\varphi_{V}\right)\;$$

(6) $$E_{Halpin-Tsai}=\frac{1+\xi \eta \varphi_{V}}{1-\eta \varphi_{V}}E_{\mathrm{PEEK}};\;\eta =\frac{E_{\mathrm{HA}}/E_{\mathrm{PEEK}}-1}{E_{\mathrm{HA}}/E_{\mathrm{PEEK}}+\xi }$$

where *E*, *E*_HA_ and *E*_PEEK_ are the elastic moduli for the composite, HA material and PEEK matrix, *φ*_V_ is the volume fraction of HA content, and the factor *ξ* is an empirical parameter determined by curve fitting of experimental data and used to quantify the geometry of the inclusion. As shown in Figure 6d, the experimental testing results were fitted using the Halpin-Tsai model, and the factor *ξ* was equal to 1.679 within the setting range of volume fraction.

**Figure 7:**
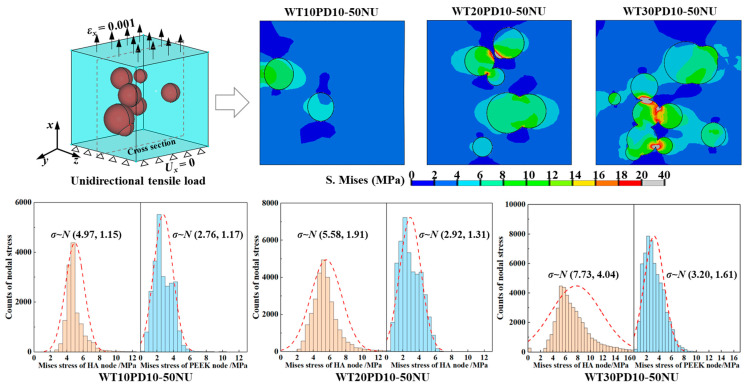
The von Mises stress distribution of composite micromodel and stress statistics of interface nodes.

The distribution of von Mises stress for three composite microscopic models under the unidirectional tensile load, and the statistical results of the von Mises stress distribution at the PEEK/HA interface nodes are shown in Figure 7. With the addition of HA, the internal stress of the PEEK/HA composite material was concentrated in the HA particles and its surrounding areas. As the HA content gradually increased, particle clustering was more likely to result in local stress concentration. By further counting the von Mises stress of the nodes on the PEEK/HA interface and using the normal distribution for statistical analysis, it was found that the stress of the nodes on the outer surface of the HA particles was generally higher than that on the contact surface with PEEK. Due to the effect of non-uniform stress distribution and local stress concentration, considering the imperfect bonding of the two materials at the interface during the preparation process, the composite is prone to interfacial stresses exceeding the bond strength during the load-bearing process, which can lead to crack initiation and propagation, as well as, ultimately, brittle fracture.

## 4. Conclusions

In this study, the mechanical properties of 3D-printed PEEK/HA composite filaments were investigated. It can be concluded that:

(1) The PEEK/HA composite filaments used for fused deposition modeling were fabricated. With an increase in HA content from 10 wt% to 30 wt%, the elastic modulus of the composite increased from 2.36 GPa to 2.79 GPa, while the tensile strength decreased from 95 MPa to 74 MPa, and the fracture elongation decreased from 63% to 23%, exhibiting brittle fracture failure under uniaxial tensile testing.
(2) The influence of particle diameter and dispersity on the elastic modulus of PEEK/HA composite was estimated using finite element analysis. When HA particles were uniformly distributed, the elastic modulus was less affected by the change in particle diameter. However, the modulus and anisotropic coefficient increased with the increase in HA content and particle clustering. Therefore, during the preparation process of the PEEK/HA composite, it is necessary to ensure the uniformity of particle size and dispersion uniformity as far as possible, so as to ensure the stability of its mechanical properties.
(3) The mathematical relationship between the elastic modulus of a composite and the volume fraction of HA content was established on the basis of the Halpin–Tsai model and experimental data. The empirical parameter *ξ* was equal to 1.679 within the setting range of volume fraction.

## Data Availability

The data presented in this study are available on request from the corresponding authors.
